# Dielectrophoretic manipulation of the mixture of isotropic and nematic liquid

**DOI:** 10.1038/ncomms8936

**Published:** 2015-08-05

**Authors:** Soo-Dong Kim, Bomi Lee, Shin-Woong Kang, Jang-Kun Song

**Affiliations:** 1 School of Electronic & Electrical Engineering, Sungkyunkwan University, Seoburo Jangan-gu, Suwon 440-746, Korea; 2 Department of BIN Convergence Technology, Chonbuk National University, Jeonju 561-756, Korea

## Abstract

In various applications involving liquid crystals, the manipulation of the nanoscale molecular assembly and microscale director alignment is highly useful. Here we show that a nematic–isotropic mixture, a unique bi-liquid system, has potential for the fabrication of microstructures having an ordered phase within a disordered phase, or *vice versa*. The volume expansion and shrinkage, migration, splitting, mergence and elongation of one phase within the other are easily accomplished via thermal treatment and dielectrophoretic manipulation. This is particularly achievable when one phase is suspended in the middle. In that case, a highly biased ordered-phase preference of surfaces, that is, the nematic-philic nature of a polyimide layer and the nematic-phobic nature of a self-assembled monolayer of chlorosilane derivatives, is used. Further, by combining this approach with photopolymerization, the patterned microstructure is solidified as a patterned polymer film having both isotropic and anisotropic molecular arrangements simultaneously, or as a template with a morphological variation.

## Abstract (web summary)

Nematic and isotropic liquid phases can coexist within a narrow temperature window. Here, Kim *et al.* show how to control this mixed phase via thermal and dielectrophoretic manipulations, based on which nematic microfilaments in an isotropic medium, or *vice versa*, are demonstrated.

## Graphical abstract

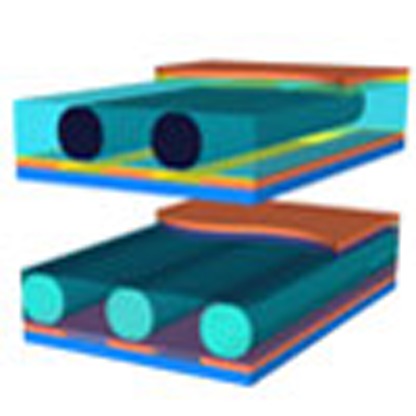

Thermotropic liquid crystal (LC), a self-assembled mesophasic material, exhibits a series of different phases depending on the temperature. According to the Landau-de Gennes theory, which elucidates the first-order transition between nematic and isotropic phases in calamitic LCs, nematic and isotropic phases can metastably coexist within a certain temperature range in a single-component LC because of the double-well-shaped free energy with respect to the order parameter^1^,^2^. This temperature range is usually less than a few degree Celsius and is influenced by the free-energy-barrier height between the two phases, the slope of the intermolecular interaction energy with respect to the temperature and the surface-confinement conditions^2^,^3^,^4^. In multi-component LCs, the two phases can stably coexist according to Gibbs' phase rule. Although the nematic and isotropic phases are both fluidic liquids and their chemical compositions are identical, the difference in their phase symmetry causes a significant disparity in their material properties^2^. These discrepancies include the elasticity; anisotropy of the optical, electrical and magnetic responses; miscibility with other materials; surface energy; and polarization. Moreover, the two phases are immiscible with each other, and hence, there is a definite boundary between one phase and another. The dispersed phase is likely to form spherical droplets within the continuous phase to obtain a minimum surface energy.

Mixtures of different types of liquids have been studied intensively, as they are of fundamental scientific interest and have high applicability for biology, electro-dynamic devices, electrophoretic devices and even LC droplets within water^5^,^6^,^7^,^8^,^9^,^10^,^11^. However, the mixed state of nematic and isotropic phases has not been regarded as a bi-liquid mixture for such applications and has instead been studied to better describe the phase transition under various conditions. This is despite the fact that a mixture of two different liquid crystalline phases can exhibit interesting and unique features^12^. For example, by tuning the temperature, one can control the volume ratios of both phases in a closed system, which is not achievable in other mixture systems^13^,^14^.

Here we shed new light on the coexisting state of isotropic and nematic phases by considering this state as a bi-liquid mixture system. We manipulate the shapes of one phase and its volume fraction to the other phase by controlling the electric field, surface type and temperature. In particular, we utilize the selective preferences of various surfaces for the nematic and isotropic phases. A highly biased preference for one phase over the other yields the disfavoured phase suspended in the middle of the favoured phase. Then, the application of a weak electric field tunes the shape or position of the suspended phase. This methodology provides a novel method utilizing two-phase LC mixtures.

## Results

### Biased preference for (dis-)ordered phases of surfaces

It is known that LC phase transitions are significantly influenced by the surface materials^4^,^15^,^16^. Usually, a good polymeric alignment layer causes LC molecules to align along a pre-set direction, which increases the transition temperature, *T*_NI_, near the surface. This process is called pre-wetting^16^,^17^. Hence, the surface can be said to favour contact with an ordered nematic phase rather than an isotropic phase. On the other hand, a self-assembled monolayer (SAM) of trichlorosilane derivatives on glass substrates favours an isotropic phase, causing surface-induced disordering, whereby near-surface LCs transit into the isotropic phase prior to the transition of the bulk LCs during the heating of the cell^12^,^18^. It was shown that the unusual surface-induced disordering phenomenon on SAMs originates from their molecular-level roughness^18^. The extremely uneven surface roughness at the molecular scale disturbs the ordering of LC molecules and induces isotropic molecular arrangement near the surface, in contrast to the polymer alignment layer. Using the nematic-philic nature of polyimide (PI) and the nematic-phobic nature of SAMs, we can produce a suspended isotropic phase surrounded by a nematic medium or *vice versa*, in the nematic–isotropic coexisting state, as explained below.

**Figure 1:**
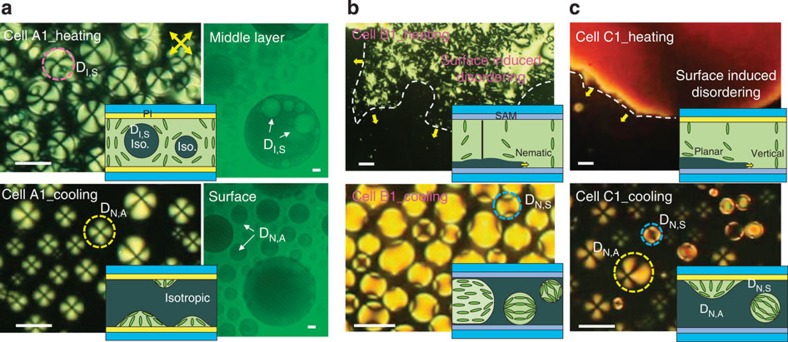
Appearance of isotropic and nematic droplets during heating and cooling. (**a**) POM images for cells with PI layers (A1), (**b**) SAMs (B1) and (**c**) heterogeneous surfaces of one PI and one SAM (C1). Here the top and bottom images correspond to the formations of the isotropic phase during heating and the nematic phase during cooling. The inset illustrations show the geometry of the corresponding droplets and the LC director profiles in each image. For all images, the polarizer and analyser directions are diagonally crossed (inset yellow arrows in **a**). The dotted magenta, yellow, and blue circles denote a suspended isotropic droplet, an anchored nematic droplet, and a suspended nematic droplet, respectively. The vertical locations of the anchored droplets (D_N,A_) and suspended droplets (D_I,S_) were confirmed by LSCM analysis (right images in **a**). Scale bars, 10 μm.

Usually, the temperature range for isotropic–nematic coexistence is far wider in multi-component LCs than in single-component LCs. For example, the temperature range was found to be <0.05 °C in a cell filled with 5CB (a typical single-compound LC), in which the coexistence arises from the metastability of the second-lowest energy-minimum phase, but it was ∼2.4 °C in a cell filled with E7 (a four-compound mixture) and ∼3.2 °C in MLC-7026 (Merck, Seoul, Korea) (a commercial mixture composed of ∼10 different components; see Supplementary Fig. 1). This may be due to the slight compositional shift that occurs in multi-component LC mixtures during the separation into the nematic and isotropic phases^19^. To obtain a stable biphasic state within a wide temperature range, we used MLC-7026 with a negative dielectric anisotropy (Δ*ɛ*) for most of our experiments. Three types of LC cells (A1, B1 and C1) were fabricated using substrates with either a PI layer for vertical alignment or an octadecyltrichlorosilane SAM: A1 had PI layers on both substrates, B1 had two SAM substrates and C1 had heterogeneous SAM substrates on the bottom substrates and PI on the top substrates. The LC cells were observed under a polarized optical microscope (POM) near *T*_NI_. Figure 1 shows the POM images taken during the heating and cooling of the cells. Here the top and bottom images correspond to the formations of the isotropic phase during heating and the nematic phase during cooling, respectively. For the A1 cell with PI layers, suspended isotropic droplets (D_I,S_) appeared following the heating of the cell above *T*_NI_, and nematic droplets anchored on the surfaces (D_N,A_) appeared during cooling from the isotropic phase to below *T*_NI_ (Fig. 1a). The vertical location of the D_I,S_ was in the middle of the LC layer, and the D_N,A_ was attached to the surfaces. Although this was roughly confirmed through POM observation by adjusting the vertical microscopic focus (see Supplementary Fig. 2), the poor quality of vertical resolution of the POM limited the clearness of the verification. Hence, we re-examined the vertical locations of droplets using laser scanning confocal microscopic (LSCM) analysis for a thicker cell, as shown in the right images in Fig. 1a. The right bottom image in Fig. 1a, which was taken from the surface, clearly indicates the D_N,A_ attached to the surface, but the right top image, which was taken from the middle of LC layer, shows D_I,S_ suspended in the middle (see Supplementary Fig. 3 for details). The LC director profiles of these droplets are illustrated in the insets of each image. The differences in the droplet geometry and vertical locations of the D_I,S_ and D_N,A_ cause different birefringence textures. The boundary lines of the D_I,S_ are clearly discernible, whereas those of the D_N,A_ are not, and the textures around the D_I,S_ are interconnected, whereas those of the D_N,A_ are independent. These observations agree well with the fact that the nematic-philic nature of PI causes isotropic droplets to be suspended rather than adhere to the surface^20^.

The cell with SAMs, B1, exhibited the opposite preference (Fig. 1b). On heating, the surface-induced disordering accompanying the anchoring transition occurred first, as shown in the upper image in Fig. 1b^12^,^18^. The anchoring transition was caused by the planar alignment of the nematic phase on the boundary with the isotropic phase, as illustrated by the director profiles in the inset, indicating the strong isotropic preference of the SAM surface. As the cell was cooled from the isotropic phase, suspended nematic droplets (D_N,S_) appeared in the middle of the LC layer. The D_N,S_ exhibited bipolar textures that differed significantly from those observed for the D_I,S_ and D_N,A_, clearly proving that the nematic droplets were not anchored on the surface. Some droplets were larger than the cell thickness, but the bipolar textures were sustained because of the thin isotropic layer near the surface. Thus, the SAM surface favours the isotropic phase, and therefore, the nematic phase is likely to form suspended droplets. After further cooling of the cell, some nematic droplets were anchored on the surface and darkened as a result of the vertical alignment.

In the cell with heterogeneous surfaces, C1 (Fig. 1c), surface-induced disordering was observed on the SAM substrate during heating, and D_N,A_ and D_N,S_ appeared simultaneously during cooling. D_N,S_ with a bipolar texture was absorbed on the PI substrate as time passed and became D_N,A_ with a four-brush texture. At the moment of the transition from D_N,S_ to D_N,A_, the droplet size increased drastically, indicating that D_N,A_ are thinner than D_N,S_ (see Supplementary Fig. 4a). The suspended droplets (D_I,S_ and D_N,S_) were easily moved by applying weak external pressure, but the anchored droplets (D_N,A_) were not (Supplementary Fig. 4b). All these observations support the concept of different droplet geometries appearing depending on the cell surface type. To exclude the possibility of the gravitational effect for the vertical position of the droplets, we performed the same microscopic observation after flipping the C1 cell on the hot stage, and practically no difference was observed, as shown in Supplementary Fig. 5. This indicates that the gravitational effect is negligible.

Thus, we confirmed the highly biased nematic preference of PI and the isotropic preference of the SAM. The important point here is that the biased preference of the surface causes isotropic droplets to be suspended within a surrounding nematic medium in A1 and nematic droplets to be suspended within the isotropic phase in B1. Even when the droplet diameter is larger than the cell thickness and the droplets are flattened by the surface confinement (for example, the D_N,S_ in Fig. 1b), the droplets can be regarded as suspended (not anchored on the surface), because the surface is likely to be wetted by the more favourable LC phase. This is confirmed by the pre-wetting on the PI layer and the surface-induced disordering on the SAM^12^,^17^.

### Dielectrophoresis for isotropic and nematic droplets

Interestingly, we electrically actuated the suspended droplets. When electric fields were applied to a cell with a partially covered electrode, the isotropic and nematic phases were repelled by and attracted to the electrode, respectively (see Supplementary Fig. 6). When a sufficiently strong electric field was applied to the nematic phase, the LCs were rearranged so that the axis with a higher dielectric constant was parallel to the electric fields. As a result, the effective dielectric constant of the nematic phase (*ɛ*_N_) along the field direction was always greater than that of the isotropic phase under the application of strong electric fields. Because of the difference between the effective dielectric constants of the two phases, the dielectrophoretic force of an isotropic droplet within the nematic phase can be expressed as^21^,^22^


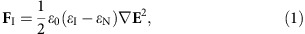


where *ɛ*_0_ is the vacuum permittivity, and generated in this way. Because *ɛ*_I_<*ɛ*_N_, **F** becomes negative, and an isotropic droplet is expelled from a region with strong electric fields. Because *ɛ*_N_=*ɛ*_⊥_ for an LC with a negative Δ*ɛ* and *ɛ*_N_=*ɛ*_||_ for an LC with a positive Δ*ɛ*, the corresponding dielectrophoretic force can be rewritten as


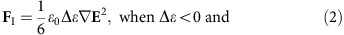



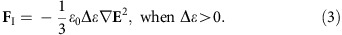


In either case, the isotropic droplet is expelled from the high-electric-field area, and as a result the electrode shape can determine the position of the isotropic and nematic phases (Supplementary Fig. 6).

### Field-induced isotropic and nematic filaments

**Figure 2:**
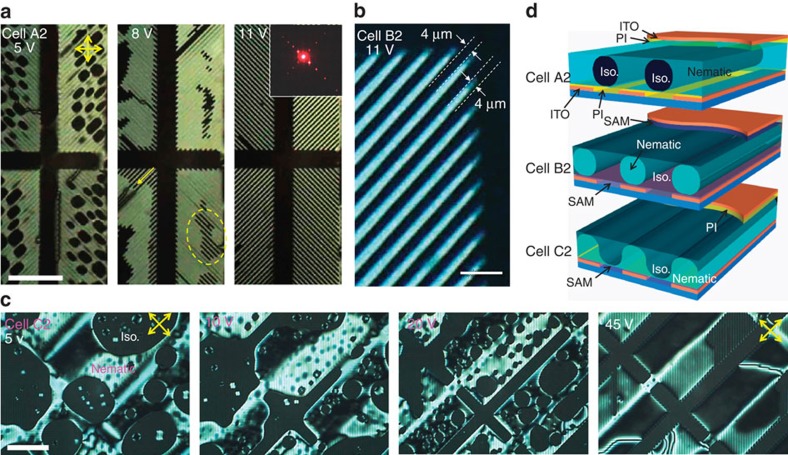
Field-induced formation of microfilaments. (**a**) In cell A2 with PI, isotropic droplets merged on the wide electrode-less channel at ∼5 V and split into small filaments (the dotted yellow circle) at 8 V. The fine filaments grew along the gap between fine electrodes (the yellow arrow) with the further increase of fields. Finally, micro-isotropic filaments were formed at 11 V. (**b**) In cell B2 with the SAM, similar microfilaments were formed. The expanded POM image shows the dimensions of the filaments. (**c**) In cell C2 with the heterogeneous alignment layers, microfilaments were not obtained, even at 45 V. (**d**) Schematic illustrations for the suspended isotropic filaments in A2 (top) and for the nematic filaments in B2 (middle) at high voltages. In cell C2, the heterogeneous alignment layers prevented the formation of filaments, but the undulation of the nematic-layer thickness may be induced by a high voltage (bottom). The crossed yellow arrows denote the directions of polarizer and analyzer. Scale bars, 100 μm for **a**,**c** and 10 μm for **b**.

Not only the position but also the shape of droplets was electrically controllable. This was particularly true when the phase was not anchored on the surface. For this test, we fabricated LC cells containing a fish-bone indium-tin-oxide (ITO) electrode with fine long electrodes 4 μm in width and electrode-less channels 30 μm in width (see Supplementary Fig. 7). To precisely investigate the dielectrophoresis with respect to the surface type, we fabricated three types of cells with the same surface layers as those in Fig. 1. In Fig. 2, cells A2 and B2 had homogeneous surfaces on PI and SAM substrates, respectively, and C2 had heterogeneous surfaces, that is, a SAM on the top and a PI layer on the bottom.

After filling A2 with MLC-7026, we carefully increased the temperature to almost *T*_NI_, at which isotropic droplets appeared. We applied 5-V electric fields (60 Hz, square wave) and the isotropic droplets began to move towards the 30-μm-wide channel. The wide channel was then primarily occupied by isotropic droplets, as shown in the first image of Fig. 2a. By increasing the applied voltage to 8 V, the isotropic droplets on the fine electrodes were split into small and long filaments along the fine electrode (the dotted circle in the second image of Fig. 2a), and isotropic filaments began to grow from the pixel edges (the yellow arrows). By further increasing the voltage to 11 V, nematic filaments 4 μm in width and over 100 μm in length completely occupied the entire fine-electrode area, as shown in the last image of Fig. 2a. A He–Ne laser beam passing through the LC cell displayed clear diffraction patterns when the nematic filaments were produced (inset image), indicating the formation of a high-quality optical grating. Similar microfilaments were generated in B2, and the required voltage for filament formation was the same as that for A2. Figure 2b shows the magnified POM image for B2, where fine filaments 4 μm in width are clearly discernible.

In contrast, cell C2, which used heterogeneous surface materials, did not exhibit any microfilaments, even up to an applied voltage of 45 V. As shown in Fig. 2c, the application of 5 V did not move the isotropic lump of the LCs, and increasing the voltage to 20 V caused some isotropic droplets to move to the wide channel in the centre. However, a large amount of isotropic droplets remained on the fine electrode. At 45 V, the majority of the isotropic phase moved to the wide-channel area, but fine microfilaments were not created, although the dielectrophoretic force must be higher than that in the other cells. As the applied field increased, the optical stripe patterns (not filaments) on the fine-electrode region became far clearer.

### Origin of the formation of microfilaments

One should note that dielectrophoretic manipulation has been primarily studied for a mixture of two liquids with largely different dielectric responses (for example, water and oil) to date, because the dielectric difference causes the driving force of the deformation of droplets^7^. However, the manipulation in the isotropic–nematic mixture requires a voltage extraordinarily lower than those required in conventional dielectrophoresis^23^, despite the small dielectric contrast between the two phases. One mechanism for this unusual phenomenon is weak interfacial tension between the isotropic and nematic phases. As the interfacial tension between two liquids increases, the restoration force to minimize the surface area of a droplet increases. In our system, the isotropic and nematic phases are chemically identical; hence, their interfacial tension is quite low, which dramatically reduces the driving voltage, as indicated in Fig. 2a.

The surface preference for the isotropic or nematic phases also influences the underlying mechanism of the phenomenon. According to the analyses of the droplets shown in Fig. 1, suspended isotropic droplets are naturally created in A2 with PI layers on both substrates, because the PI layer disfavours the isotropic phase. Thus, the isotropic droplets are separated from the surfaces. The suspended isotropic droplets are easily controlled using the weak dielectrophoretic force, because there is no surface frictional force. Hence, the isotropic droplets are extended by the dielectrophoretic force and form finely striped filaments. A schematic illustration of the isotropic filaments in A2 is shown at the top of Fig. 2d. Similarly, suspended nematic droplets are formed within B2 having SAMs and, as a result, the microfilaments in B2 are nematic filaments, as illustrated in the middle of Fig. 2d. On the other hand, in C2, which has a SAM on the top surface and a PI layer on the bottom substrate, the isotropic phase is likely to attach to the top substrate, and the nematic phase is likely to attach to the bottom substrate. Hence, a wide-layered structure is more likely to form than thin filaments. As shown at the bottom of Fig. 2d, a nematic layer with an undulated thickness may be obtainable at a high field, as indicated in Fig. 2c, where the optical stripe patterns become clearer in the fine-electrode region as the applied voltage increases.

### Formation of microfilaments during heating and cooling

**Figure 3:**
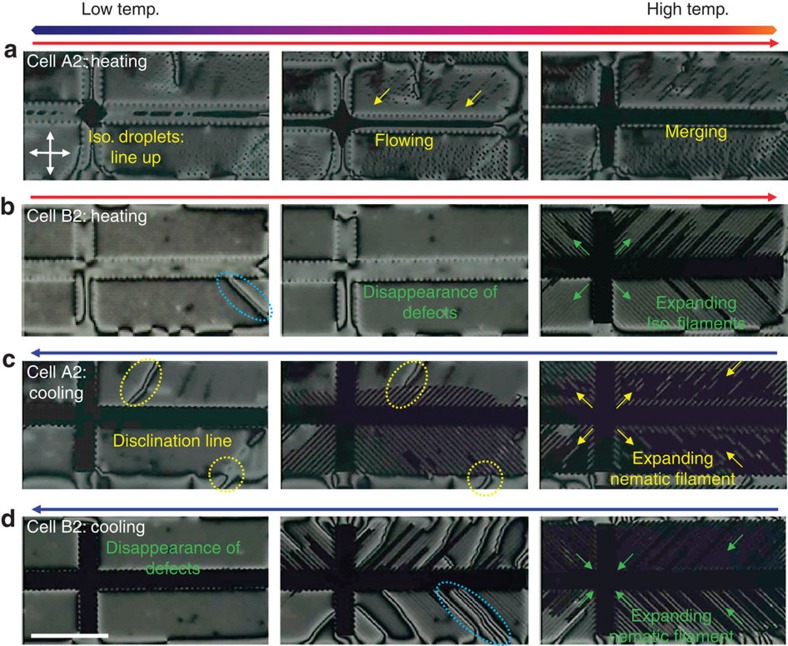
Microfilaments on heating and cooling at a fixed voltage. (**a**) Microfilament formation during heating of A2 and (**b**) B2. Isotropic droplets can align, flow (the yellow arrows), merge and expand in A2. (**c**) Microfilament formation during cooling of A2 and (**d**) B2. The disclination lines automatically disappear in B2 (the dotted blue circles), whereas those in A2 (the dotted yellow circles) are sustained. The yellow arrows in **c** denote the expanding nematic filaments, and the crossed arrows in **a** denote the directions of polarizer and analyzer. Scale bar, 100 μm.

Under a fixed applied voltage (15 V), the temperature in A2 and B2 was precisely controlled. During the heating of A2, small isotropic droplets randomly appeared and aligned along the gap between the two neighbouring fine electrodes (the first image in Fig. 3a). Some of the droplets flowed steadily along the fine electrode towards the wide channel (the second image), and others expanded and merged to form a long filament (the last image). The isotropic-droplet drift indicates that there was no surface anchoring of the isotropic droplets in A2.

On the other hand, during the heating of B2, surface-induced disordering was observed prior to the filament formation. This was confirmed by the disappearance of the disclination lines (Fig. 3b), which was caused by the weakened azimuthal anchoring for the nematic phase due to the presence of thin isotropic layer near the surface. Following further heating, a large isotropic region appeared in the wide channel, and fine isotropic filaments expanded along the gaps between the fine electrodes.

When the LC cells were cooling, the nematic phase sporadically appeared and the small nematic filaments expanded gradually; this was observed in both A2 and B2 (Fig. 3c,d). The nematic filaments merged to form a wide nematic film on further cooling. During the merging of the filaments, disclination lines appeared as a result of the topological mismatch between the neighbouring filaments. The disclination lines for A2 were fixed (Fig. 3c), but those for B2 soon disappeared (Fig. 3d). This indicates that the nematic film in A2 was anchored on the surface, but that in B2 was suspended on the isotropic surface. All these observations agree well with the interpretation based on the selective preferences of the individual surfaces.

### Microfilaments of a single-compound LC with positive Δ*ɛ*

**Figure 4:**
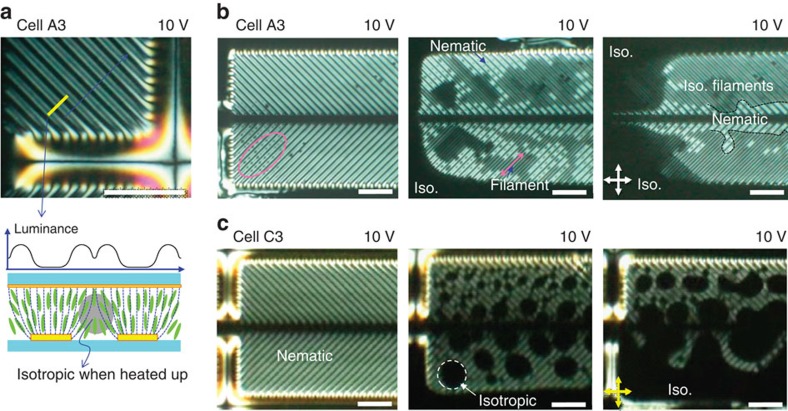
Microfilaments using 5CB with positive dielectric anisotropy. (**a**) A POM image of cell A3 with 5CB under the application of an electric field and corresponding director profile and luminance profile. Dark lines appear on top of the fine electrode, and thinner dark lines appear between the two electrodes. (**b**) Microfilament formation during heating of A3. (**c**) No microfilaments were formed in C3. The crossed arrows denote the directions of polarizer and analyzer. Scale bars, 50 μm.

In the previous section, the dielectrophoretic manipulation in a two-phase mixture was demonstrated using a multi-compound LC mixture with a negative Δ*ɛ*. Such manipulation is also applicable for other types of LCs, including single-compound LCs and LCs with a positive Δ*ɛ*, because these LCs are also subjected to the nematic-philic or nematic-phobic property of surfaces. To confirm this, we fabricated filaments using 5CB, which has a positive Δ*ɛ*. We prepared two types of cells: one with PI layers on both substrates (A3) and the other with a PI layer on the substrate with fine ITO electrodes and a SAM on the opposite substrate (C3). Under the application of 10 V, the temperature of the cells was precisely controlled. As shown in Fig. 4a, in contrast to those of the cells with MLC-7026, the POM image of A3, with 5CB, showed clear dark and bright stripe patterns along the fine electrodes, even in the nematic phase. The directors aligned parallel to the field direction because 5CB has a positive Δ*ɛ*. Thus, the area on top of the electrodes appeared dark, the edge of the electrodes was the brightest. Another thin dark stripe appeared in the centre of the gap between two adjacent fine electrodes, as illustrated in the director profile across the fine electrodes and the corresponding luminance profile in Fig. 4a.

As we heated A3 to around *T*_NI_, small isotropic droplets appeared along the gap between the fine electrodes (in the magenta circle in Fig. 4b), and the isotropic droplets merged and expanded to form long stripes (the magenta arrow in Fig. 4b). Eventually, the most of fine-electrode area was filled with fine isotropic filaments, except in the nematic phase at the centre of the pixel and in the isotropic phase outside of the pixel. The location of the isotropic filament is shown in Fig. 4a. In contrast, the birefringence pattern of C3 was the same as that of A3 in the nematic phase (Fig. 4c), but the isotropic droplets were not elongated but instead formed circular domains at *T*_NI_. Increasing the temperature further caused the isotropic droplets to merge, filling the whole area.

Thus, the overall dielectrophoretic behaviours are identical to those of the cells with MLC-7026, except for the optically half-pitched stripes due to the vertical alignment of the 5CB with a positive Δ*ɛ*, in the narrower temperature range where filaments appear.

### Polymerized isotropic and nematic mixed patterns

**Figure 5:**
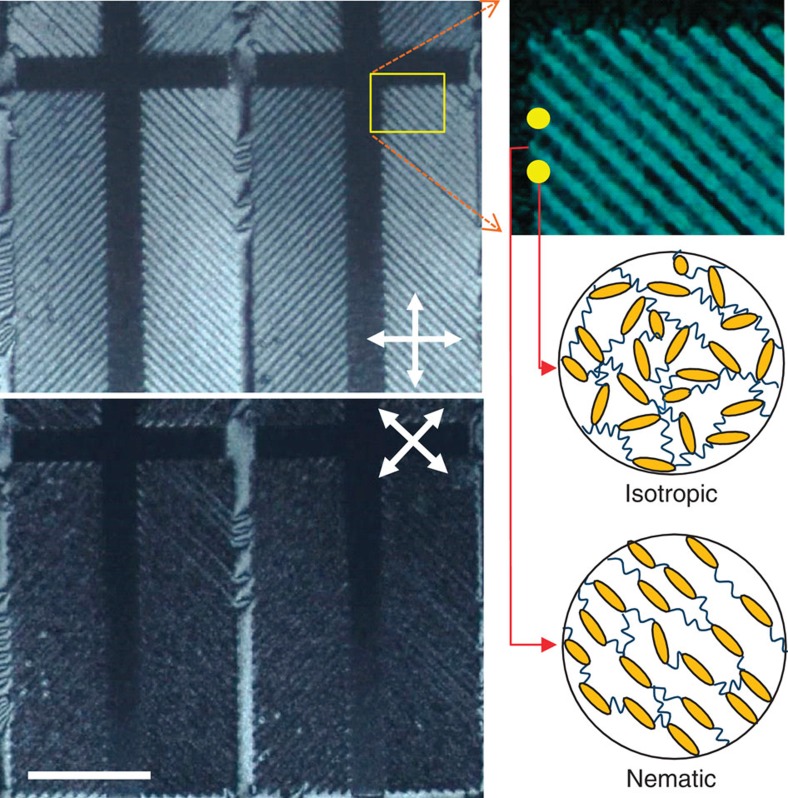
Polarized optical patterns of polymerized filament film. POM images with fish-bone-slits aligned 45° and parallel, respectively, to the crossed polarizers, confirming the optical pattern of the polymer film with alternating nematic and isotropic order. The inset arrows denote the orientations of the polarizer and analyser. Expanded filaments (top right) and corresponding illustrations for the completely disordered mesogen state and the uniaxial order of the mesogens in the film plane. The crossed arrows denote the directions of polarizer and analyzer. Scale bar, 100 μm.

The isotropic filaments formed in the nematic LC were solidified using photo-polymerizable LC monomers. The nematic mixture was prepared by adding 50 wt% of reactive mesogen with a photo-initiator (UCL-001-K1, Dainippon Ink and Chemicals, Japan) into MLC-7026. The mixture was then loaded into A2 to induce isotropic filaments in the nematic host, as illustrated in Fig. 3a. Under the application of electric fields, the cell was exposed to ultraviolet light with a peak wavelength of 367 nm using a mercury lamp (100-W power, 3,000-lm typical luminous flux, Osram, Germany) equipped within a fluorescence microscope (E600, Nikon, Japan). The polarized optical pattern with alternating isotropic–nematic phases was completely frozen into the solidified polymer networks after ultraviolet exposure for a sufficient time. The solidified isotropic–nematic patterns remained even after cooling to room temperature, as shown in the POM images of Fig. 5, which were taken under different directions of crossed polarizers (see the white arrows) at room temperature. The optical images precisely matched the patterns observed prior to the polymerization. This strongly indicates the order and disorder of the polymer networks at a molecular level, as illustrated in Fig. 5. The interchanging nematic and isotropic orders, with the fish-bone-patterned electrode as a template, evidently solidified into the polymer networks. Interestingly, the nematic director (that is, the uniaxial optic axis) was approximately aligned along the slit direction in the substrate plane. The dark regions in the two POM images in Fig. 5 (inter-slit area and wide channels) correspond to the optically isotropic and molecularly disordered polymer. The illustrations in Fig. 5 denote the molecular orientation of the networks at the corresponding regions. This clearly demonstrates that the isotropic filaments formed in the nematic LC can act as a template that can be used to imprint isotropic–nematic molecular orders into patterned biphasic polymer networks.

### Morphological difference in isotropic and nematic films

**Figure 6:**
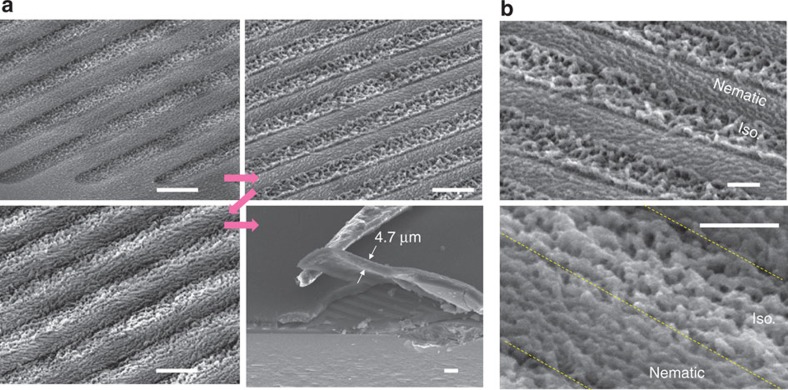
Morphological evolution during polymerization process. (**a**) The SEM images show the sequential evolution of the patterned polymer film with increasing ultraviolet exposure time (the thick magenta arrows denote the sequence). Randomly clustered and orientationally ordered clusters grow separately in the inter-electrode and electrode regions, respectively. After full ultraviolet irradiation, a solid uniform film with a thickness of 4.7 μm, which is the same as the cell thickness, was fabricated. Scale bars, 5 μm. (**b**) (Top) A clear morphological contrast was observed at the initial stage of the polymerization. The nematic region had ordered and relatively even polymer clusters, whereas the isotropic region had disordered, porous clusters at the micrometre scale. (Bottom) As the polymerization process progressed, the morphological difference was reduced, but the ordered clusters in the nematic region and the porous clusters in the isotropic region were still clearly observed. Scale bars, 2 μm.

The ultraviolet exposure time was intentionally adjusted to gradually increase from one part of the cell to the other. The cell was dismantled, and unreacted LC was preferentially removed by soaking in ethanol for 1 day and then analysed using a scanning electron microscope (SEM). The SEM images further elucidate the microscopic morphologies that developed during the polymerization process of the reactive LCs. Figure 6a shows representative SEM images of the patterned networks observed from different locations with varying ultraviolet exposure time. As soon as the polymerization is initiated, the phase separation of the polymer aggregates occurs in both the nematic and isotropic phases, and the polymer clusters grow in separate regions. Polymerization occurring concurrently in the different phases results in morphologically distinct polymer cluster structures, as shown in the figures. As indicated by Fig. 6a, random polymer clusters are formed in the inter-electrode area (that is, in the isotropic phase), and no in-plane order of polymer clusters is observed. However, orientationally ordered polymer bundles in the plane of the substrate along the slit direction developed in the area of the electrode (that is, in the nematic phase). The polymer clusters continue to grow, and a morphological pattern becomes more obvious, as shown in the second and third images in Fig. 6a. The film increases in thickness as the polymerization proceeds, finally creating a thick film corresponding to the cell thickness, as shown in the last image in Fig. 6a.

The magnified SEM images shown in Fig. 6b clearly exhibit the morphological difference between the nematic and isotropic regions in the early and middle stages of the polymerization process, respectively. In the early stage (the top image in Fig. 6b), the polymer network that formed in the nematic region produced relatively dense and smooth clusters that were uniaxially aligned along the fine electrode. However, the polymer network that formed in the isotropic region was largely uneven, more porous and randomly clustered with no in-plane order. As the polymerization progresses further (the bottom image), the morphological difference between the two regions decreased, but the porously clustered isotropic polymer was clearly indicated in the isotropic region. It is interesting to note that the nanometre-scale molecular ordering of the host molecules significantly influenced the polymerization process and the cluster structure at the micrometre scale, as evidenced by uniaxially aligned dense and even polymer clusters in the nematic region and randomly distributed porous and rough polymer clusters in the isotropic host region. Thus, the coexisting nematic–isotropic coexistence pattern is a template for not only optically birefringent–isotropic patterns at the molecular scale but also the ordered–disordered or smooth–rough surface morphology at the micrometre scale.

## Discussion

We demonstrate that an immiscible mixture of ordered nematic and disordered isotropic phases can provide an interesting bi-liquid system. Although the molecular compositions of these two phases are practically identical, the different molecular arrangements cause contrasting wettability on the PI and SAM surfaces and lead to a dielectrophoretic-driving ability. In particular, the highly biased wettability of the PI and SAM results in the formation of suspended isotropic droplets in a cell with PI layers and suspended nematic droplets in a cell with SAMs. When a droplet is suspended within another phase, we can easily expand, elongate, transfer, merge and split the droplets by dielectrophoresis and the thermal propagation of the phase boundary. This approach allows us to fabricate isotropic microfilaments in a nematic medium and nematic microfilaments in an isotropic medium. The electric field required for inducing the microfilaments is extraordinarily low compared with that needed by conventional electro wetting devices. The filament shapes can be precisely controlled according to the shapes of the electrodes designed on the substrates, and the molecular alignment within the microfilaments is influenced by the boundary confinement. Thus, patterned biphasic polymer networks with alternating isotropic–nematic molecular orders are successfully obtained using a method based on the combined one-step polymerization of a photo-reactive monomer.

## Methods

### Fabrication of cells for droplet-formation observation

A commercial LC mixture, MLC-7026, was used in the experiments. It had a negative dielectric anisotropy (Δ*ɛ*=−3.9), *T*_NI_ of 75 °C and birefringence (Δ*n*) of 0.083 at room temperature. A PI substrate was obtained by spin-coating a commercial vertical-alignment PI solution, AL60101 (JSR, Japan), on bare glass substrates, which were then baked at 230 °C for 1 h. A SAM was obtained by dipping a clean glass substrate in an octadecyltrichlorosilane (Aldrich Korea, Korea)–toluene solution for 30 min. The LCs were vertically aligned on the PI and SAM substrates and planar on the ITO substrate. This was experimentally confirmed (the results are not shown here). An LC droplet was dropped on the bottom substrate and then covered by another substrate. The thickness of the LC layer was in the order of several tens of micrometres, and the temperature was controlled using a laboratory-made hot stage for POM observation.

### Fabrication of cells with fish-bone electrodes

A fish-bone electrode was embedded on the bottom substrate only (Supplementary Fig. 7), and the upper substrate was fully covered by an ITO electrode. The fish-bone electrode comprised many rectangular pixels 200 × 540 μm^2^ in size, and each pixel had fine branch-electrodes 4-μm in width, with a 4-μm distance between neighbouring slits extending in four directions. A 30-μm-wide channel that was not covered by an electrode was located in the centre of each pixel. The substrates were coated with either PI or a SAM, and the cell thickness was 3.5 μm, which was sustained by ball spacers.

### LSCM observation

A rod-shaped green fluorescence dye, cumarine-6, was added to the MLC-7026 mixture at 0.01 wt%. The dye-doped LC was injected in a cell with PI layers on both substrates, and the LC layer thickness was 100 μm. An erected-type LSCM, K1-Fluo, manufactured by nanoscope systems in Korea was used. The pin-hole was set to 0.5 a.u., and a 405-nm laser beam was used. An objective with a magnitude of × 20 and a numerical aperture of 0.65 was used.

## Additional information

**How to cite this article:** Kim, S.-D. *et al.* Dielectrophoretic manipulation of the mixture of isotropic and nematic liquid. *Nat. Commun.* 6:7936 doi: 10.1038/ncomms8936 (2015).

## Supplementary Material

Supplementary Information Supplementary Figures 1-7
