# Supplementary material for: Dielectrophoretic manipulation of the mixture of isotropic and nematic liquid
Source: Nat Commun. 2015 Aug 5;6:7936. doi: 10.1038/ncomms8936 (PMC4918331; doi:10.1038/ncomms8936)
Supplement: Supplementary Information — Supplementary Figures 1-7 [file ncomms8936-s1.pdf]

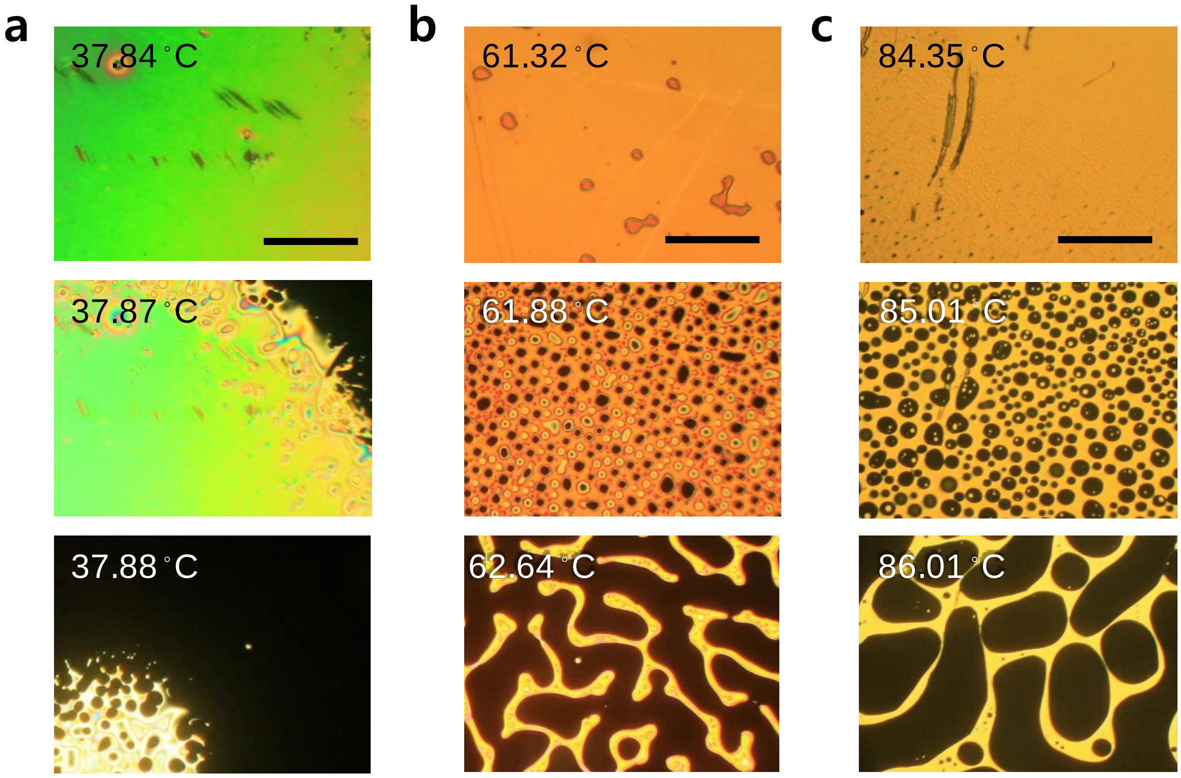

Supplementary Figure 1 | Temperature window for isotropic and nematic coexistence. The temperature window depends sensitively on the number of compounds in the liquid crystal (LC) mixtures. The microscopic photos were taken during the heating of a cell, which comprised two bare glass substrates and was filled with various LCs. (**a**) A 5CB cell (single-compound) exhibited only 0.01 and 0.05 °C windows during heating and cooling, respectively (mean: 0.03 °C); (**b**) an E7 cell, which is a mixture of 5CB, 7CB, 8OCB, and 5CT, exhibited a 2.35 °C window during heating and a 2.53 °C one during cooling (mean: 2.44 °C); and (**c**) a MLC-7026 cell, which is a commercial mixture supplied by the Merck company, exhibited a 2.92 °C window during heating and a 3.40 °C one during cooling (mean: 3.16 °C). The thicknesses of these cells were roughly 5–15 μm. Scale bars, 100 μm.

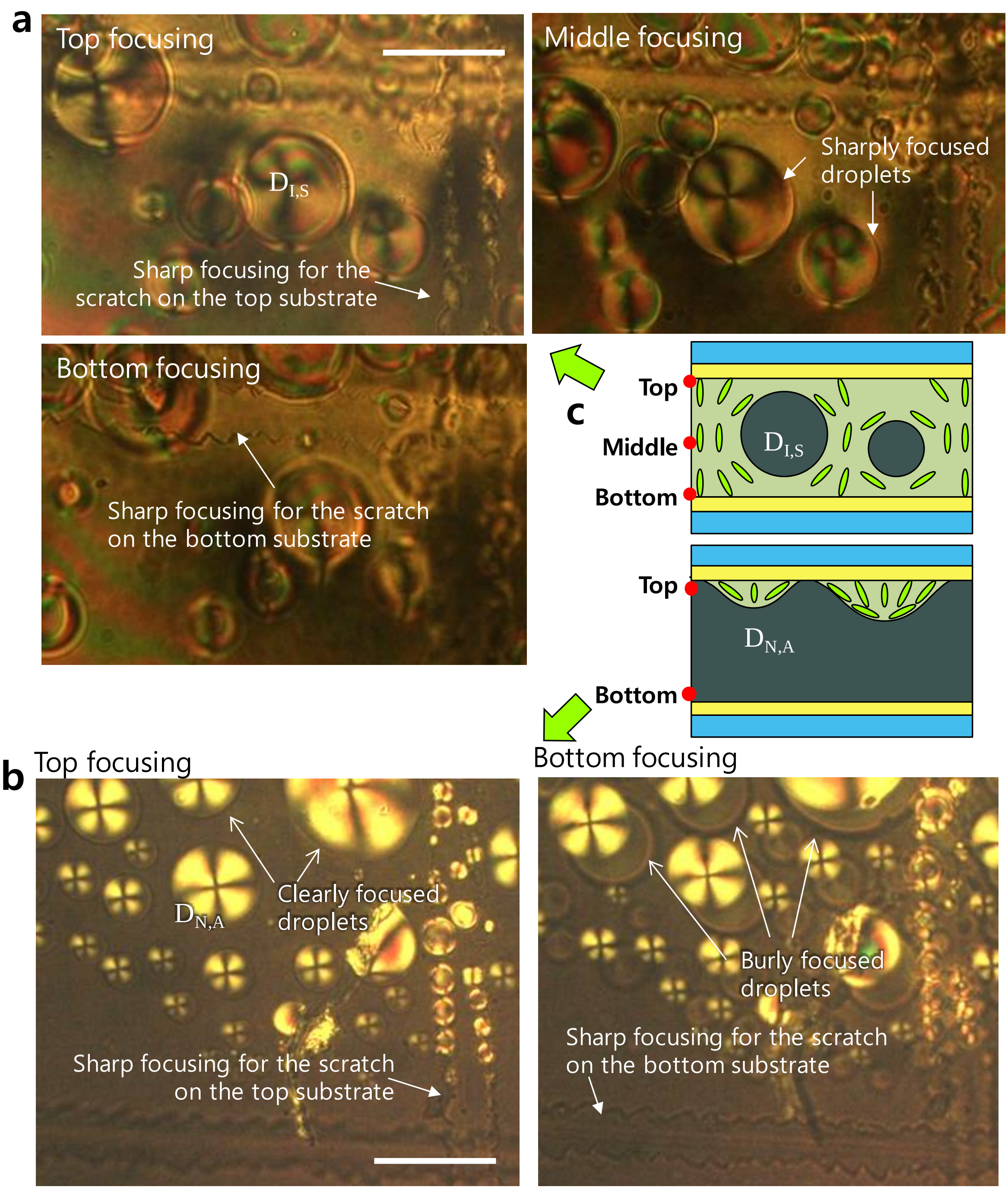

Supplementary Figure 2 | Vertical positions for $D_{I,S}$ and $D_{N,A}$ droplets in A1. (**a**) For the $D_{I,S}$ droplets, the clearest droplet boundary was obtained when the vertical focusing was adjusted to the middle of the LC layer. Scratches were intentionally made vertically and horizontally on the top and bottom substrates, respectively, to discriminate between the vertical focusing positions in the POM observation. The $D_{I,S}$ droplet boundary line was blurred for the top and bottom focusing, but the droplet boundary became clear in the middle focusing of the LC layer, indicating that the droplet was located at this position. (**b**) For $D_{N,A}$ droplets, the clearest image was obtained for top focusing, indicating that the $D_{N,A}$ droplets were located on the surface. (**c**) Illustrations for the vertical positions of droplets in **a** and **b**, respectively. The cell thickness was ~60 μm. Scale bars, 20 μm.

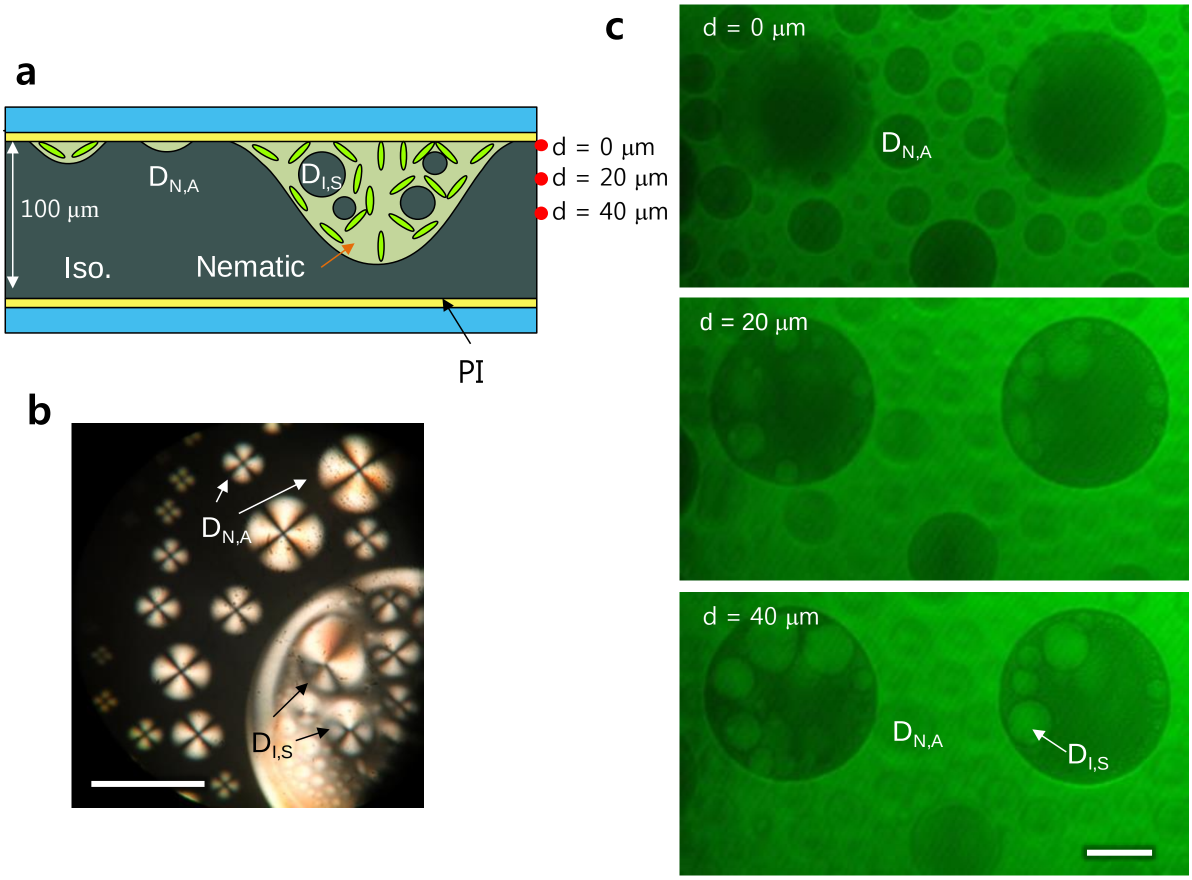

Supplementary Figure 3 | Vertical positions for $D_{I,S}$ and $D_{N,A}$, observed using LSCM. (**a**) Illustration of cell geometry and droplets. A 100-μm-thick cell was used to clearly visualize the vertical positions of the droplets. (**b**) POM image of the cell, showing a large nematic droplet at the right bottom containing suspended isotropic droplets. (**c**) LSCM images for different vertical position. An objective of ×20 magnitude and NA of 0.65 was used. The top image was taken from the surface focusing, which clearly indicates the $D_{N,A}$ droplets. The middle and bottom images were taken at depths of 20 and 40 μm, respectively, from the surface. As the depth increased, the boundary lines for $D_{I,S}$ became clearer, and those for $D_{N,A}$ became blurred. This clearly verifies that the $D_{N,A}$s are on the surface and that the $D_{N,A}$s are in the middle layer. Scale bars, 50 μm.

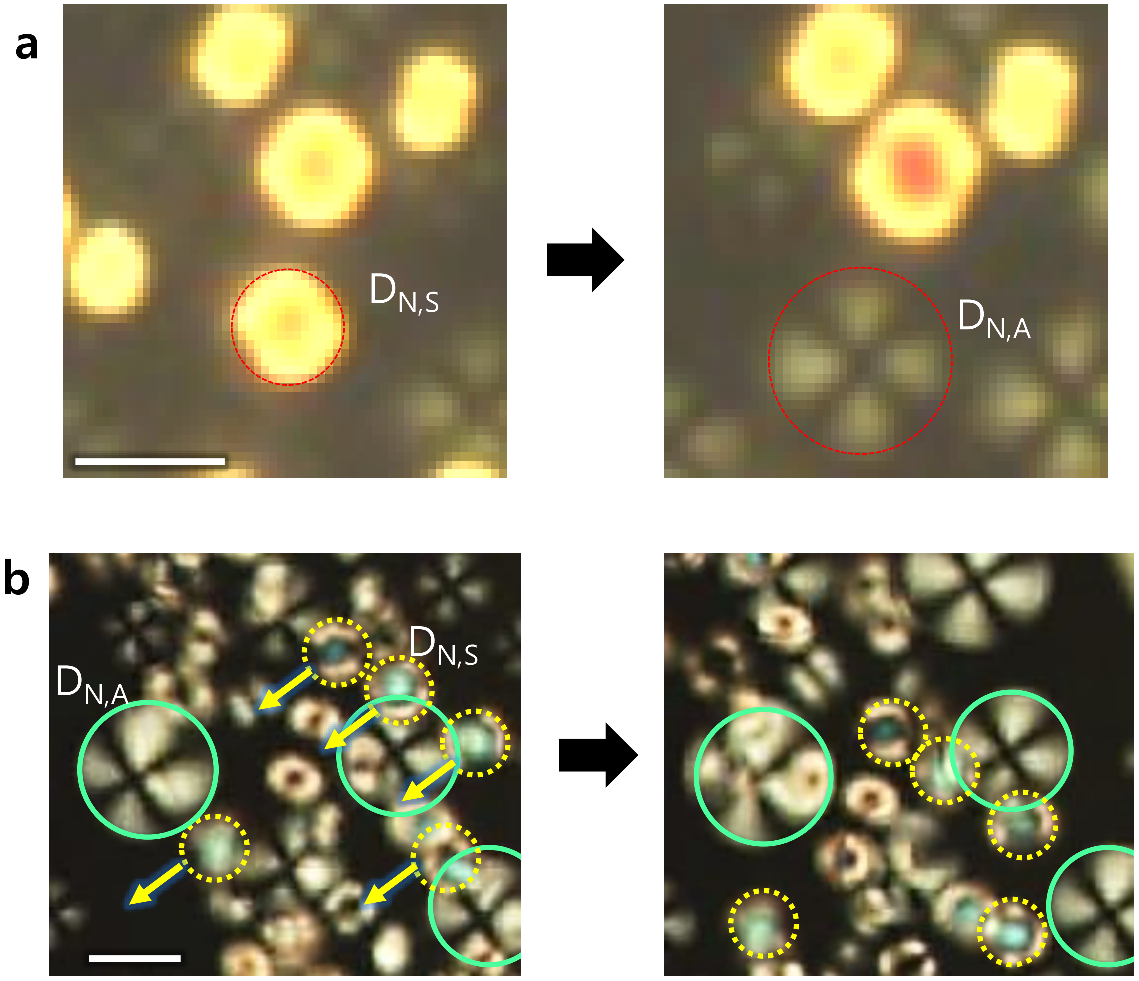

Supplementary Figure 4 | Transition from a suspended to an anchored droplet, and flowing droplets. (**a**) In a heterogeneous cell (C1), a suspended droplet with bipolar texture ($D_{N,S}$ in the left image) was absorbed on the PI substrate as time passed and became an anchored droplet with a four-brush texture ($D_{N,A}$ in the right image). At the moment of the transition from $D_{N,S}$ to $D_{N,A}$, the droplet size increased drastically, indicating that $D_{N,A}$ droplets are thinner than $D_{N,S}$ droplets. (**b**) When the cell was weakly pressed, an LC flow appeared. The suspended droplets ($D_{N,S}$) were easily moved, but the anchored droplets ($D_{N,A}$) were immobile, indicating their adhesion to the surface. Scale bars, 20 μm.

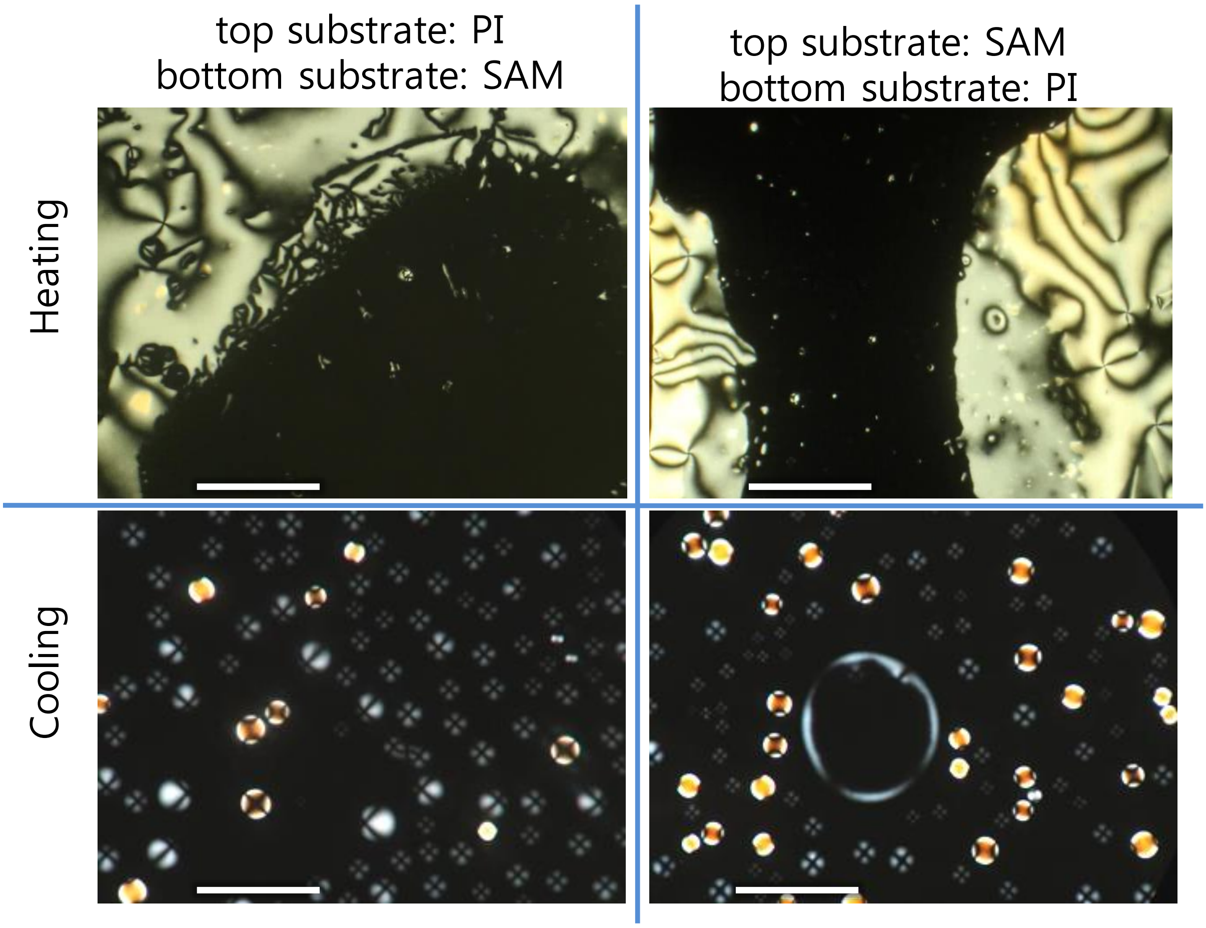

Supplementary Figure 5 | Gravitational effect for the shape of droplets. First, a microscopic observation was made during heating and cooling for a cell with a PI layer on the top substrate and an SAM layer on the bottom substrate (left images). Then, the cell was flipped, and the same experiment was performed (right images). No difference was observed, indicating that the gravitational effect is negligible in our study. Scale bars, 100 μm.

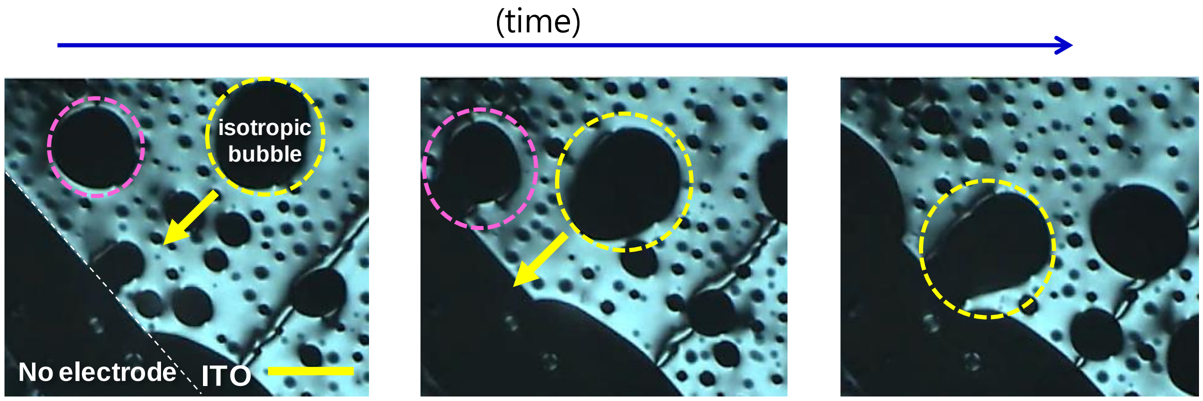

Supplementary Figure 6 | Dielectrophoresis of isotropic and nematic droplets. When a 10-V 60-Hz electric field was applied to the cell, the isotropic droplets gathered in the electrode-free area, and the nematic droplets gathered in the area with electrodes. As shown in the above microscopic images, the electrode-free area was primarily filled with the isotropic phase, and newly generated isotropic droplets in the electrode area slowly moved to the electrode-free area. Scale bars, 30 μm.

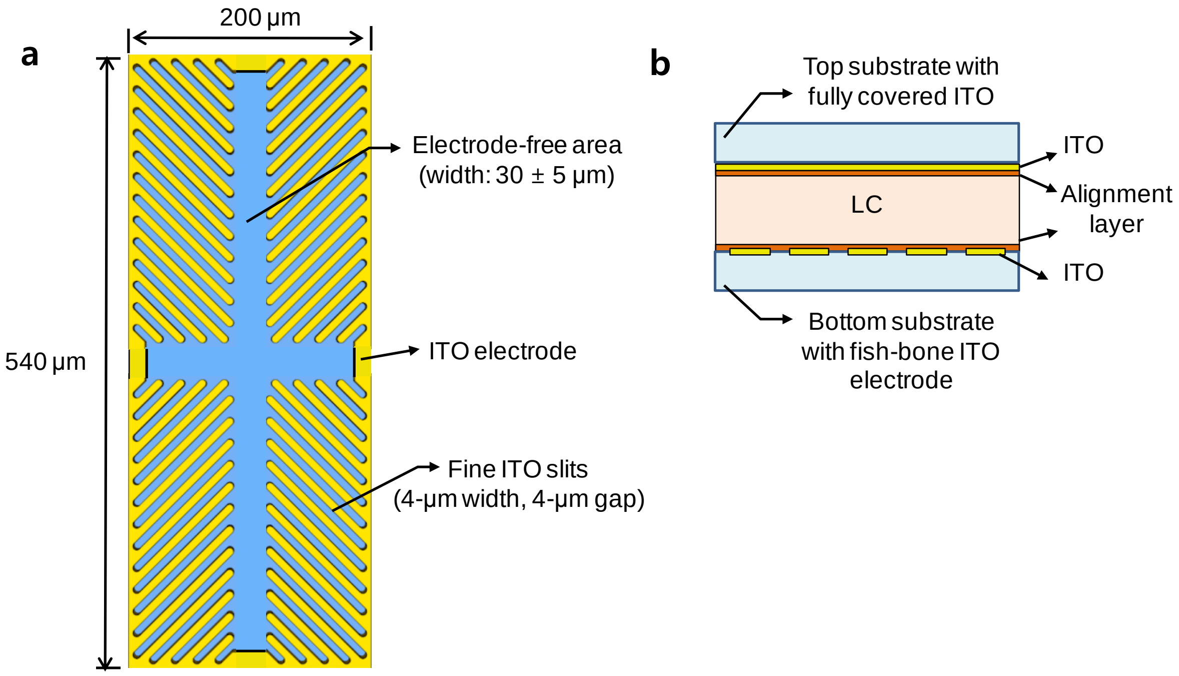

Supplementary Figure 7 | Schematics of pixel and cell structures used for microfilament fabrication. (**a**) The bottom substrate has a fish-bone-shaped ITO electrode, and the fine-slit ITO electrodes are 4 µm in width and have a 4-µm distance between neighbouring slits. In the centre, there is a wide electrode-free area ~30 µm wide. One pixel unit has a width and height of 200 and 540 µm, respectively. (**b**) A cell was made using the fish-bone-shaped ITO substrate on the bottom side. The top substrate had a fully covered ITO electrode, and the alignment layer was composed of PI or SAM.
